# Cytokinin-Specific Glycosyltransferases Possess Different Roles in Cytokinin Homeostasis Maintenance

**DOI:** 10.3389/fpls.2016.01264

**Published:** 2016-08-23

**Authors:** Mária Šmehilová, Jana Dobrůšková, Ondřej Novák, Tomáš Takáč, Petr Galuszka

**Affiliations:** ^1^Department of Molecular Biology, Centre of the Region Haná for Biotechnological and Agricultural Research, Faculty of Science, Palacký University in OlomoucOlomouc, Czech Republic; ^2^Laboratory of Growth Regulators and Department of Chemical Biology and Genetics, Centre of the Region Haná for Biotechnological and Agricultural Research, Faculty of Science, Palacký University in Olomouc and Institute of Experimental Botany ASCROlomouc, Czech Republic; ^3^Department of Cell Biology, Centre of the Region Haná for Biotechnological and Agricultural Research, Faculty of Science, Palacký University in OlomoucOlomouc, Czech Republic

**Keywords:** cytokinin, glycosyltransferase, *Arabidopsis*, senescence, GFP subcellular localization

## Abstract

Plant hormones cytokinins (CKs) are one of the major mediators of physiological responses throughout plant life span. Therefore, a proper homeostasis is maintained by regulation of their active levels. Besides degradation, CKs are deactivated by uridine diphosphate glycosyltransferases (UGTs). Physiologically, CKs active levels decline in senescing organs, providing a signal to nutrients that a shift to reproductive tissues has begun. In this work, we show CK glucosides distribution in *Arabidopsis* leaves during major developmental transition phases. Besides continuous accumulation of *N*-glucosides we detected sharp maximum of the glucosides in senescence. This is caused prevalently by *N*7-glucosides followed by *N*9-glucosides and specifically also by *trans*-zeatin-*O*-glucoside (*t*ZOG). Interestingly, we observed a similar trend in response to exogenously applied CK. In *Arabidopsis*, only three UGTs deactivate CKs *in vivo*: UGT76C1, UGT76C2 and UGT85A1. We thereby show that *UGT85A1* is specifically expressed in senescent leaves whereas *UGT76C2* is activated rapidly in response to exogenously applied CK. To shed more light on the UGTs physiological roles, we performed a comparative study on UGTs loss-of-function mutants, characterizing a true *ugt85a1-1* loss-of-function mutant for the first time. Although no altered phenotype was detected under standard condition we observed reduced chlorophyll degradation with increased anthocyanin accumulation in our experiment on detached leaves accompanied by senescence and stress related genes modulated expression. Among the mutants, *ugt76c2* possessed extremely diminished CK *N*-glucosides levels whereas *ugt76c1* showed some specificity toward *cis*-zeatin (*c*Z). Besides *t*ZOG, a broader range of CK glucosides was decreased in *ugt85a1-1.* Performing CK metabolism gene expression profiling, we revealed that activation of CK degradation pathway serves as a general regulatory mechanism of disturbed CK homeostasis followed by decreased CK signaling in all UGT mutants. In contrast, a specific regulation of *CKX7*, *CKX1* and *CKX2* was observed for each individual UGT mutant isoform after exogenous CK uptake. Employing an *in silico* prediction we proposed cytosolic localization of UGT76C1 and UGT76C2, that we further confirmed by GFP tagging of UGT76C2. Integrating all the results, we therefore hypothesize that UGTs possess different physiological roles in *Arabidopsis* and serve as a fine-tuning mechanism of active CK levels in cytosol.

## Introduction

Cytokinin (CK) homeostasis is tightly and precisely regulated in plant cells, enabling adequate response to distinct developmental and environmental requirements. CK metabolism is provided by a variety of enzymes that ensure proper hormone level maintenance. One of such regulatory enzymes is a uridine diphosphate glycosyltransferase (UGT; EC 2.4.1.) that deactivates the molecule of CK by its conjugation with a sugar moiety, mostly glucose; at *O*- and *N*- position (Entsch and Letham, 1979; Entsch et al., 1979; Martin et al., 1999; Mok et al., 2000a). Therefore, CKs can form various types of glycosides that can possess different roles. Whereas CK *N*-glucosides are thought to be terminal products of the irreversible deactivation or a detoxification pathway (McGaw et al., 1984; McGaw and Horgan, 1985; Blagoeva et al., 2004; Sakakibara, 2006; Bairu et al., 2011), CK *O*-glucosides were shown to be reversibly deglycosylated by β-glucosidase action (Brzobohatý et al., 1993; Falk and Rask, 1995; Kristoffersen et al., 2000) and therefore are thought to serve as inactive storage forms of CKs. UGTs belong to a large multi-gene family (Mackenzie et al., 1997) that deactivates a wide range of molecules. Within the last years, a molecular approach has been used to elucidate the function of the CK-specific glycosyltransferases. Mutant lines overexpressing genes for CK *O*-glycosyltransferases in maize (Pineda Rodó et al., 2008), tobacco (Mok et al., 2000a,b; Martin et al., 2001; Havlová et al., 2008), rice (Kudo et al., 2010) and *Arabidopsis* (Hou et al., 2004; Wang et al., 2011, 2013; Jin et al., 2013; Li et al., 2015) have been well characterized to date. Experiments utilizing a *c*Z *O*-glucosyltransferase gene form *Phaseolus lunatu*s L. as the overexpressed transgene have confirmed increased accumulation of *O*-glucosides in different tissues. In certain conditions, these experiments have confirmed more or less severe phenotypic alteration (Mok et al., 2000a; Martin et al., 2001; Havlová et al., 2008; Pineda Rodó et al., 2008; Kudo et al., 2010).

Up to now, CK-specific UGTs have been shown to be expressed to various levels in different tissues such as developing seeds, roots and leaves of young seedling or maturing embryo (Martin et al., 1999; Veach et al., 2003; Wang et al., 2011, 2013; Jin et al., 2013) with low expression in vegetative tissues (Martin et al., 1999). On a subcellular level, no signal sequence or transmembrane domain was found in any of the plant UGTs (Li et al., 2001) investigated so far. However, it was suggested that the proteins may be associated as peripheral components of the endomembrane system (Winkel-Shirley, 1999). Glycosylation alters chemical properties of the inactivated molecules enabling their inter-compartment re-localization driven by newly gained increased hydrophilicity (Bowles et al., 2005). This raises a question whether inactive CK glucosides are destined to retargeting to cellular compartments or whether they serve as long-distance transport forms, which is more likely, since high levels of glucosides were found in xylem sap of cucumber (Kato et al., 2002) or extracellular space of *Arabidopsis* and barley leaves (Jiskrová et al., 2016). This hypothesis was further supported by Kato et al. (2002) whose research documented shoot greening mediated by CK glucosides uptake by roots. In contrast to speculative localization of CK-specific UGTs in vacuoles (Meek et al., 2008; Pineda Rodó et al., 2008; Bajguz and Piotrowska, 2009), the *trans*-zeatin *O*-glycosyltransferase enzyme from *Zea mays* and *Phaseolus vulgaris* was immunodetected in the nucleus, cytosol, and closely associated with the plasma membrane and in the cell wall of *Z. mays* root cells (Li et al., 2001). Further, GFP tagged UGT85A1 from *Arabidopsis* has been up to now detect in cytosol, and nucleus (Jin et al., 2013). Although dual subcellular localization was observed in plant UGTs (Hong et al., 2001), this was an exceptional case. Besides the cited works, only little is known about localization of the CK glucosides on the subcellular level. Although CK *O*-glucosides were observed to be present within vacuoles of *Chenopodium rubrum* (Fusseder and Ziegler, 1988; Mok et al., 1992), our recent work shows predominant localization of both types of CK glucosides in extracellular space (Jiskrová et al., 2016).

As previous reviewers summarized, the same UGT can recognizes multiple substrates *in vitro* and, conversely, different UGTs can glycosylate the same substrate (Lim and Bowles, 2004). However, since this does not reflect physiological functions of UGTs *in planta*, identification of their mutants were shown to be the most powerful tool to address their direct roles (Bowles et al., 2005). In *Arabidopsis*, five CK-specific UGTs (UGT73C1, UGT73C5, UGT76C1, UGT76C2, UGT85A1) were biochemically characterized in the past using recombinant proteins (Hou et al., 2004). Although three of them were further confirmed to be specific toward CKs *in planta* (Wang et al., 2011, 2013; Jin et al., 2013; Li et al., 2015), UGT73C5 was shown to be specific toward brassinosteroids (BR) (Poppenberger et al., 2005) and UGT73C1 was shown to be specific to trinitrotoluene compounds (Gandia-Herrero et al., 2008) with much higher affinity than to CKs. Amongst the three CK-specific UGTs, increased sensitivity to exogenously applied CK was detected in *ugt76c2* and *ugt76c1*mutant (Wang et al., 2011, 2013) as a result of impaired CK glucosylation but only *ugt76c2* resulted in a modified phenotype manifested by smaller seeds (Wang et al., 2011). Enhanced root elongation was observed in *UGT85A1* overexpressing line (Jin et al., 2013) as a result of accelerated CK deactivation. Former studies showed that UGT76C1 and UGT76C2 are *N*-glucosylation specific (Hou et al., 2004; Wang et al., 2011, 2013) whilst UGT85A1 was proposed to be *trans*-zeatin-specific *O*-glucosyltransferase (Jin et al., 2013). Further experiments on UGT76C2 mutants demonstrated involvement of the CK deactivation pathway during drought and osmotic stress and. Moreover, these experiments also indicated temporal importance of CK glucosylation process under the stress conditions (Li et al., 2015).

The present study is aimed to extend the knowledge of the CK deactivation pathway. We take a multidisciplinary approach combining molecular biology and quantitative analysis to answer some intriguing questions regarding physiological relevance of CK glucosides and the significance of the glycosylation process in *Arabidopsis*. In this work, we describe CK glucosides formation during main developmental transition phases and further, we perform a comparative study of loss-of-function mutants of CK-specific *UGTs* to elucidate their roles in CK homeostasis maintenance during plant development and in response to exogenous stimuli. We also characterize *ugt85a1* mutant in context to CK for the first time and discuss UGT85A1 ability to deactivate a broader range of substrates as well as its specificity in senescence process. Finally, our research also attempts to bring more light into CK metabolism compartmentation in this work.

## Materials and Methods

### Plant Materials

*Arabidopsis thaliana* ecotype Columbia-0 was used in this work. Seeds of *ugt76c2* (SALK 135793C), *ugt76c1* (SALK 144355C), *ugt85A1-1* (SALK 085809C), and *ugt85a1-2* (SALK 146306C) were obtained from the European *Arabidopsis* Stock Center (for the description of the lines see Supplementary Table S1). Surface sterilized seeds were sown on half strength MS medium (Murashige and Skoog, 1962) supplemented with 1% sucrose and stratified at 4°C for 4 days in the dark prior to germination. Seedlings were grown either on MS plates or in soil under standard *Arabidopsis* growth condition in an environmental chamber (16 h fluorescence light of 150 μmol photons^∗^m^-2∗^s^-1^ intensity/8 h dark, 22°C, 55% relative humidity). A green mature fully expanded leaf (sixth and seventh) from a 4-week-old rosette was detached for experiments with exogenously applied CK and further for gene expression profiling. The leaves were incubated in water containing 10 μM KIN, 6-benzylaminopurine (BAP), isopentenyladenine (iP) or *trans*-zeatin (*t*Z) with final concentration of dimethylsulfoxid (DMSO) 0.05% for 3 days under the same conditions as the donor plants. Further, intact whole 4-week-old rosettes were sprayed with 10 μM solution of *t*Z containing 0.005% surfactant Silweet L-77 every 24 h for 3 days. In both cases, mock treatment contained 0.05% DMSO. *Solanum lycopersicum* L., Peto 343 was used for overexpression of *SU:UGT76C2-GFP* for the subcellular localization study described below.

### Identification of T-DNA Insertion Mutants

Although *UGT85A1* mutant was described before, its expression was not abolished completely (Carviel et al., 2009). In this work, we characterize a new T-DNA insertion mutant of *UGT85A1* (SALK_146306C), labeled as *ugt85a1-1*, and compare it with the previously characterized SALK_085809C (*ugt85a1-2*). Supplementary Figures S1A,C illustrates schematic of T-DNA positions within the *UGT85A1* gene. The *ugt85a1-1* and *ugt85a1-2* lines were confirmed for their T-DNA insertion position, homozygosity (data not shown) and gene expression level in RT-PCR (Supplementary Figures S1B,D). Our results showed that *UGT85A1* was expressed in WT but not in the *ugt85a1-1* mutant, while a weak expression was detected in *ugt85a1-2* mutant. Remaining loss-of-function mutants of CK-specific UGTs used in this work were analyzed for presence of their T-DNA insertion and its effect in previous publications: *ugt76c2* (Wang et al., 2011), *ugt76c1* (Wang et al., 2013). No redundancy effect of any of the CK-specific UGTs was detected in any of the mutants used in this work based on their gene expression quantification (Supplementary Figure S2).

### Phenotyping and Root Growth Assay

Sterilized seeds of *Arabidopsis* wild-type (WT) and *ugt85A1-1* were transferred to vertical square Petri dishes on a solid MS medium containing 0.5% MES and 1% sucrose as well as various concentrations of BAP, iP, dihydrozeatin (DHZ) and *t*Z, respectively. 0.05% DMSO was used in control (mock treatment) since that was the final concentration in all CK treatments. After stratification, the seeds were germinated and grown in an environmental chamber for 14 days. Within the growing period the length of the primary root was evaluated after seven and 14 days respectively, using Scion Image software (Scion Corporation, Frederick, MD, USA). The number of fully emerged lateral roots was scored under a SMZ800 Stereoscopic Microscope (Nikon, Japan). For a senescence induced experiment, sixth leaves from 5-week-old WT and *ugt85A1-1* rosettes were detached from ten individual plants for each genotype. The experiment was performed in two replicates.

### Chlorophyll and Anthocyanin Content Assay

Chlorophyll (Chl) content was performed according to published protocol (Chory, 1991) with the following modification. After 4 days of incubation under continuous light, the detached leaves were frozen in liquid nitrogen and homogenized to extract Chl in 80% acetone. Chl *a* nad *b* portions of total Chl (expressed as relative amounts) were calculated using following equation: *a* = 12.7 (A_663_) – (A_645_) and *b* = 22.9 (A_645_) – 4.68 (A_663_). Further, the same samples were used for anthocyanin content measurement which was performed according to a published protocol (Neff and Chory, 1998) using acidified methanol and counting the relative content by subtracting A_657_ from A_530_.

### *In silico* Prediction of Subcellular Localization

To predict the subcellular localization of the UGTs, the following prediction web based tools were used: TargetP (Emanuelsson et al., 2000), ProtComp v9.0^1^, SignalP 4.0 (Petersen et al., 2011), ChloroP 1.1 (Emanuelsson et al., 1999), WoLF PSORT^2^, iPSORT (Bannai et al., 2002), Plant-mPLoc (Chou and Shen, 2010).

### Cytokinin Content Determination

The procedure used for CKs purification was performed according to the described method (Svačinová et al., 2012) with subsequent modifications. The samples were extracted in modified Bieleski buffer (methanol/water/formic acid, 15/4/1, v/v/v) and then purified using two solid phase extraction columns, a C18 octadecylsilica-based column (500 mg of reversed-phase sorbent; Applied Separations) and, after that, an Oasis MCX column (30 mg of mixed-mode sorbent with reversed-phase/cation-exchange properties; Waters) (Dobrev and Kamínek, 2002). The samples were analyzed employing ultra-high performance liquid chromatography (Acquity UPLC system; Waters), coupled to a triple quadrupole mass spectrometer (Xevo TQ-S; Waters) equipped with an electrospray interface. Deuterium-labeled CK internal standards (OlChemIm) were used to validate the determination, each at 0.5 pmol per sample (Novák et al., 2008). Quantification was achieved by multiple reaction monitoring of [M + H]^+^ and the appropriate product ion. The quantification was performed by Masslynx software (v4.1; Waters) using a standard isotope dilution method. The ratio of endogenous CK to the appropriate labeled standard was determined and further used to quantify the level of endogenous compounds in the original extract according to the known quantity of the added internal standard.

### RT-PCR and Quantitative RT-PCR (qPCR) Assay

Plant material was processed for reverse transcription according to the method described in our former publication (Vyroubalová et al., 2009). The primers used in our experiment amplified specifically for the following genes: glucosyltransferases *UGT73C1*, *UGT73C5*, *UGT76C1*, *UGT76C2*, *UGT85A1*; CK dehydrogenases *CKX1*-*7*; isopentenyltransferases *IPT1-9*; CK nucleoside 5′-monophosphate phosphoribohydrolase *LOG2*, *LOG8*; CK *trans*-hydroxylase *CYP735A2*; response regulators type A (*ARR5*, *ARR15*, *ARR16*), type B (*ARR1*, *ARR2*, *ARR10*, *ARR14*); CK receptors *AHK4*, *AHK2*, *AHK3*. All the listed primers were designed according to our previously published work (Mik et al., 2011; Motte et al., 2013; also see Supplementary material of these publications for details), Rubisco small chain *RUBsc* (Hensel et al., 1993), cysteine protease senescence-associated gene *SAG12* (Gepstein et al., 2003; Wagstaff et al., 2009) and chlorophyll *a/b* binding protein *CAB2* (Kim et al., 2006), for transcription factors *MYB2* (Guo and Gan, 2011) and *WRKY53* (Hinderhofer and Zentgraf, 2001; Balazadeh et al., 2008); for 1-aminocyklopropan-1-carboxylate synthase *ACS8* as a key regulatory enzyme in the biosynthesis of the plant hormone ethylene (Tian et al., 2009); for chalcone flavanone isomerase *CHI* as anthocyanins biosynthetic enzyme (Catala et al., 2011); 9-*cis*-epoxycarotenoid dioxygenase *NCED3*, a key enzyme in the biosynthesis of abscisic acid (Tan et al., 2003). RNA from three biological replicates was transcribed in at least two independent reactions and each cDNA sample was run in at least three technical replications on Viia7^TM^ Real-Time PCR System in a default program (Life Technologies). To evaluate a relative quantification of analyzed genes, cycle threshold (C_t_) values were analyzed with the Formula method (Livak and Schmittgen, 2001) and normalized with respect to actin 2 (*ACT2*) and small nuclear ribonucleoprotein D1 (*SnRNPD1*) (Quilliam et al., 2006) that were used as internal standards. The expression data are relative quantities (RQ) calculated as 2^-ΔCq^ extrapolated to controls that are given as 1.0. To confirm UGT85A1 expression level of *ugt85a1* mutants, 85A1fw and 85A1rev primers were used (Carviel et al., 2009) in RT-PCR.

### Cloning and Generation of GFP Translational Fusions

A Modular Binary Construct System with AKK1436 as a shuttle vector and AKK1472B binary vector (Christopher Taylor Lab, Donald Danforth Plant Science Center, St. Louis, MO, USA) was used for GFP gene fusion and subsequent overexpression of the GFP tagged *UGT76C2* gene in tomato hairy roots under a super ubiquitin (*SU*) promoter as described in our previous work (Šmehilová et al., 2009). The gene for UGT76C2 was synthetized using GeneArt^®^ commercial service (Thermo Fisher Scientific, USA) with At5g05860 CDS as a template sequence without any modifications. *Bam*HI restriction sites were added to the sequence to enable further sub-cloning of the synthetic gene from the source pMK-RQ vector. Use of *BamH*I digestion and further ligation to AKK1436 vector resulted in in-frame UGT76C2-GFP fusion further incorporated into AKK1472B through *Pac*I digest/ligation.

### Transgenic Tissue Preparation and Analysis

Root transformation was performed as described previously (Collier et al., 2005). The binary constructs containing *SU:UGT76C2-GFP* and *SU:GFP* were introduced into *Agrobacterium rhizogenes* strain 15834, verified for transgenes presence and further used for transgenic tomato hairy roots production following the procedure used in our previous work (Šmehilová et al., 2009).

### Subcellular Localization of GFP-Fused Proteins

The transgenic roots were first evaluated for GFP fluorescence with a SMZ800 Stereoscopic Microscope (Nikon) and analyzed for transgene expression in RT-PCR. Three to five individual roots were selected from the confirmed lines and mounted in 100 mM phosphate buffered saline (PBS) pH 6.5 prior to observation in confocal microscopy. Zeiss LSM710 laser-scanning confocal microscope (Zeiss) was used to detect the GFP signal of the *SU:UGT76C2-GFP* and *SU:GFP*. GFP was excited at 488nm and detected between 500 and 535 nm. Images were processed using Zeiss ZEN software and Adobe Photoshop software.

## Results

### Cytokinin *N*7-Glucosides Accumulate during Whole Plant Development, Whereas *O*-Glucosides Fluctuate with Sharp Maximum of *t*ZOG in Senescent Leaf

**FIGURE 1:**
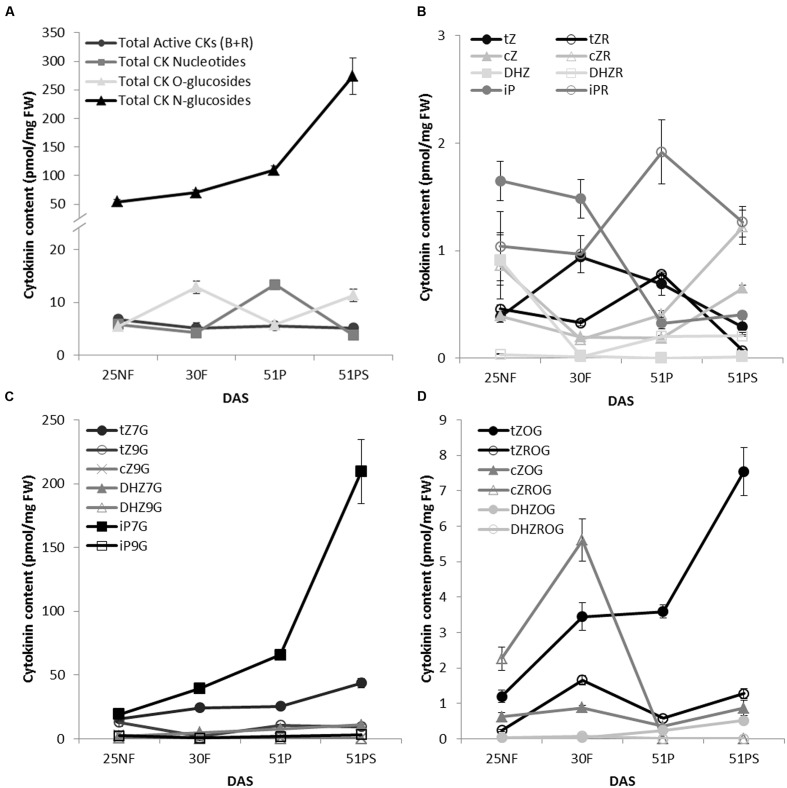
**Cytokinin content of WT *A. thaliana* leaves during transitions between vegetative, reproductive and senescence stages.** The time points were: green fully developed leaves from a non-flowering (NF) rosette, a flowering (F) rosette, from a plant with maturing pods (P); and senescent leaves from the same plant (PS); days after sowing (DAS). The overall distribution of cytokinins grouped by types of their forms **(A)** and with a focus on individual forms: active cytokinins **(B)**, cytokinin *N*-glucosides **(C)** and cytokinin *O*-glucosides **(D)**. Values are the means of three biological replicates ± SD.

To determine CK glucosylation status during *Arabidopsis* leaf senescence, levels of isoprenoid CKs and their respective ribosides, ribotides and glucosides were determined under standard growth conditions in four time points covering the main developmental transition phases: green mature (fully expanded) leaves of non-flowering and flowering rosettes, and green and senescent leaves from pod forming plants. Generally, *N*-glucosides accumulated during leaf aging (**Figure 1A**) with sharp maximum of *N*7**-glucosides in yellowing stage of the leaf aging (**Figure 1C**). It might seem that CK *O*-glucosides fluctuated in an opposite way to CK nucleotides when summed up (**Figure 1A**); however, **Figure 1D** illustrates flowering time-related increase of all *O*-glucosides except DHZOG and *t*ZOG that accumulated in aging-dependent manner. Our results point out that *t*ZOG is the main *O*-glucoside present in *Arabidopsis* senescent leaves since its level arose almost seven times in comparison to other CK *O*-glucosides in this time point. Indeed, we observed a decline in active CK forms (**Figure 1A**), with fluctuation characteristic for each form (**Figure 1B**). Interestingly, *c*Z and particularly *c*ZR levels increased in senescent leaf which is in accordance with previous work (Gajdošová et al., 2011). It should be noted that the content of CK glucosides was more than one order of magnitude higher than active forms.

### Gene Expression Profile of Cytokinin-Specific UGTs

**FIGURE 2:**
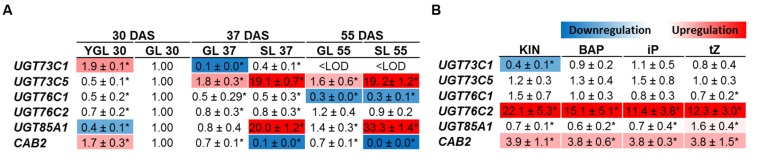
**Gene expression profile in leaves of Arabidopsis cytokinin-specific *UGTs* during plant development (A) and after cytokinin treatment (B).** YGL, Young green leaf 30 DAS; GL, green fully developed leaf; SL, senescent leaf. In the experiment where transcript level in response to exogenously applied cytokinin was analyzed, green mature fully expanded leaves (sixth and seventh) from 4 weeks old rosette were used and treated by 10 μM cytokinins for 3 days. The gene expressions are expressed as relative quantities and extrapolated relative to the GL 30 DAS **(A)** and mock treatment **(B)**, respectively, given as 1.0. The values represent means of three biological replicates (three technical replicates) with standard deviations. Asterisks indicate significant differences between the controls and the samples according to unpaired Student’s *t*-test, ^∗^*P* < 0.05; <LOD, below limit of detection.

Up to date, the UGTs expression was assessed only in young developing tissues (Wang et al., 2011, 2013). According to our data, *Arabidopsis* accumulated large quantities of CK glucosides in leaves in age-dependent manner so that we employed gene expression profiling to determine which UGTs are specific in CK deactivation process during senescence. We show that the most upregulated CK-specific UGT was *UGT85A1* whose expression is specifically boosted in senescent leaves (**Figure 2A**) followed by BR-specific *UGT73C5* (Poppenberger et al., 2005). We detected significantly downregulated expression of *UGT76C1* with age-dependent manner but without specificity in yellowing leaf. Steady expression was detected for UGT76C2 throughout the developmental stages, whereas varying expression was detected for *UGT73C1*. *CAB2* gene was used as a control of senescence progress that reflects photosynthesis status. Consistently, downregulated expression of *CAB2* in senescent leaves correlates with previously published data (Kim et al., 2006).

To compare UGTs in their response to various exogenously applied CK we analyzed modulation of *UGTs* expression in detached leaves in response to 10 μM KIN, BAP, iP and *t*Z, respectively. Generally, no significant difference in transcript level was detected in all but *UGT76C2* whose transcript level was increased enormously in response to all the tested CKs, with the highest increase after KIN uptake (**Figure 2B**) in comparison to mock treatment. *UGT85A1* was sensitive only to *t*Z treatment which increased its expression only mildly, 1.59-times. Surprisingly, the second CK *N*-specific *UGT76C1* glycosyltransferase showed no response to any of the CKs on transcript level.

### Cytokinin Content after Exogenous Application of Cytokinins

**FIGURE 3:**
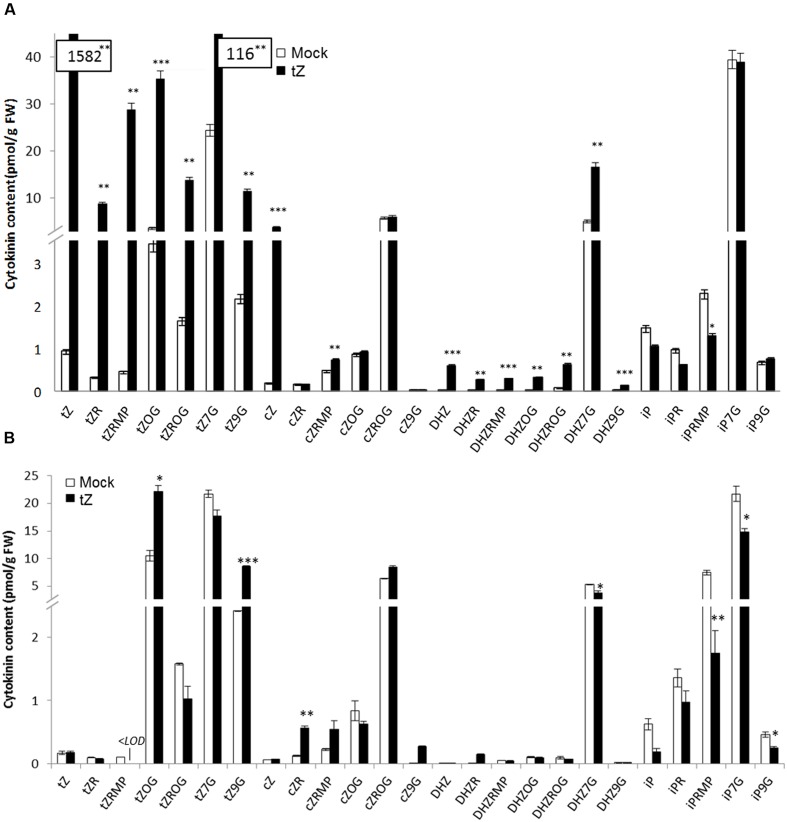
**Cytokinin content in whole plants of WT *A. thaliana* after *t*Z treatment (A) and in detached leaves after KIN treatment (B).** Values are the means of three biological replicates with ± error bars that represent standard deviations. Asterisks over bars indicate significant differences between KIN treated and mock control plants according to two-tailed unpaired Student’s *t*-test, ^∗^*P* < 0.05, ^∗∗^*P* < 0.01, ^∗∗∗^*P* < 0.001.

In order to correlate increased transcript level of *UGT76C2* after exogenously applied CK, we determined CK content in detached leaves after KIN treatment (as a non-native *Arabidopsis* CK not interfering with natural CK content) and in whole 4-week-old rosettes in response to *t*Z. Subsequently, the most prevalent CK types (Mok and Mok, 2001) and forms were analyzed. After *t*Z uptake, a strong interfering effect in terms of extremely elevated *t*Z-type CKs was observed (**Figure 3A**). Further, all DHZ-type CKs arose as well. Consistently with previous studies (Cowley et al., 1978; Letham et al., 1978; Hou et al., 2004), *N*7**-glucosides were the predominant forms of CK deactivation pathway, since strong increase of *t*Z7G and DHZ7G were detected after *t*Z uptake, followed by its *O*-glucosides. *t*Z9G and *t*ZOG were the most significantly elevated CKs when leaves were treated by KIN (**Figure 3B**). Both treatments resulted in abolished CK biosynthesis as manifested by general decline of all iP-type CKs and particularly of *t*ZRMP in case of KIN treatment. Interestingly, *c*Z level rose after *t*Z uptake, whereas no change was detected in KIN experiment. Since KIN does not interfere with native *Arabidopsis* CK content but triggers the same CK response in *Arabidopsis* (Mik et al., 2011; Motte et al., 2013), we used KIN to study CK-mediated response in UGT mutants.

### *ugt85a1-1* Loss-of-Function Mutant Shows Delayed Senescence Phenotype under Stress Condition

**FIGURE 4:**
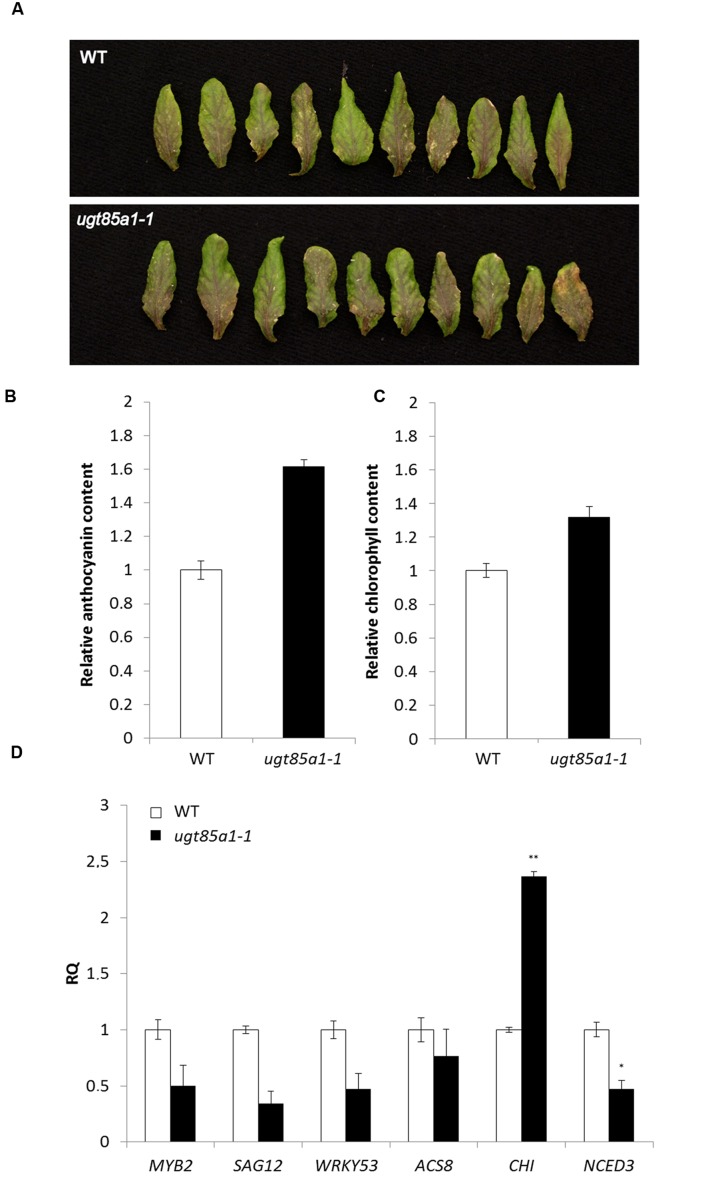
**Characterization of *ugt85a1-1* mutant.** Detached leaves of WT and the mutant after 3 days of continuous light **(A)**, chlorophyll content **(B)**, anthocyanin content **(C)** and gene expression profile of senescence and stress related markers **(D)** including anthocyanin biosynthetic gene *CHI*. Values are the means of two independent experiments where leaves of ten individual plants were pooled and expressed as relative quantities extrapolated to WT control (white) that is given as 1.0 with error bars representing standard deviations. Asterisks indicate significant difference between WT and transgenic tissue according to two-tailed unpaired Student’s *t*-test, ^∗^*P* < 0.05, ^∗∗^*P* < 0.01.

In this study, UGT85A1 was shown on transcript level to be the major form responsible for CK inactivation during senescence. However, the *ugt85a1-1* mutant did not show apparent phenotypic alteration under normal growth conditions. In order to investigate expected enhanced sensitivity of *ugt85a1-1* mutant to exogenously applied CK, a rooting test was performed using BAP, iP and *t*Z in 0.1, 0.5, and 1.0 μM concentrations. No significant difference in main root length or in number of lateral roots was observed after any of the treatments. Since no phenotype difference was observed on whole plant level in any of developmental stages analyzed, we performed an experiment where senescence was induced by leaves detachment. After 3 days of incubation, the leaves of *ugt85a1-1* mutant showed apparent enhanced accumulation of anthocyanins (**Figure 4A**). This was further verified by the anthocyanin assay that confirmed 1.6-fold increase of anthocyanins in *ugt85a1-1* (**Figure 4B**). Similarly, chlorophyll content was measured showing reduced chlorophyll degradation in *ugt85a1-1* mutant (**Figure 4C**). In order to show senescence status of the mutant in this experiment, we determined gene expression of senescence and stress associated genes together with genes responsible for anthocyanin biosynthesis *CHI* (**Figure 4D**). Relative expression of all the senescence and stress marker genes was lower in *ugt85a1-1* mutant in comparison to the WT control with significant reduction of gene transcript of the key enzyme responsible for abscisic acid biosynthesis *NCED3*. Higher relative expression of *CHI* gene coding for one of the core anthocyanin biosynthetic enzymes further supports observed enhanced anthocyanin accumulation. Taken together, our data suggest important role of UGT85A1 in senescence process.

### *UGT85A1* Can Be Specific toward a Broader Range of Substrates

**FIGURE 5:**
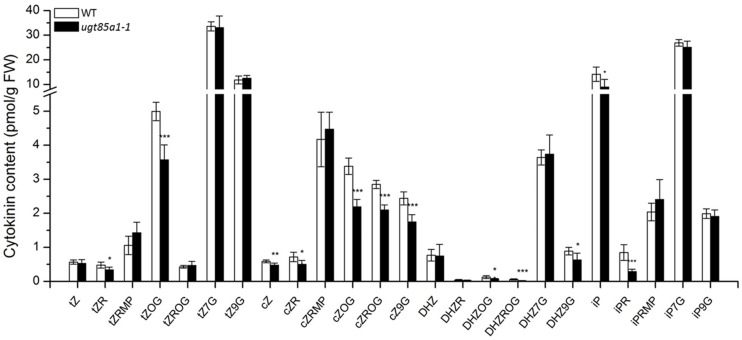
**Cytokinin content of 4-week-old seedlings of *ugt85a1-1* mutant.** Values are the means of three biological replicates with error bars representing standard deviations. Asterisks over bars indicate significant difference between wild-type and the mutant according to two-tailed unpaired Student’s *t*-test, ^∗^*P* < 0.05, ^∗∗^*P* < 0.01, ^∗∗∗^*P* < 0.001.

We determined CK profile of *ugt85a1-1* in 25-day-old seedlings. As indicated in **Figure 5**, our data show significant decrease in *t*ZOG level that is in accordance with previously published data where UGT85A1 was proposed to specifically *O*-glucosylate *t*Z based on *UGT85A1* overexpressor characterization (Jin et al., 2013). Our data further point out that this UGT might possibly glucosylate also other CKs since significantly decreased levels of almost all *O*-glucosides as well as *c*Z9G and DHZ9G were detected. However, this could be due to other uncharacterized regulatory processes that occur in the mutant. Although some of the active CKs decreased, particularly iP, iPR, *c*Z, *c*ZR, and *t*ZR, CK nucleotides somewhat increased. No change in any of *N*7**-glucosides was observed in our data.

### Comparison of Cytokinin Content in Detached Leaves of UGT Loss-of-Function Mutants after KIN Treatment

**FIGURE 6:**
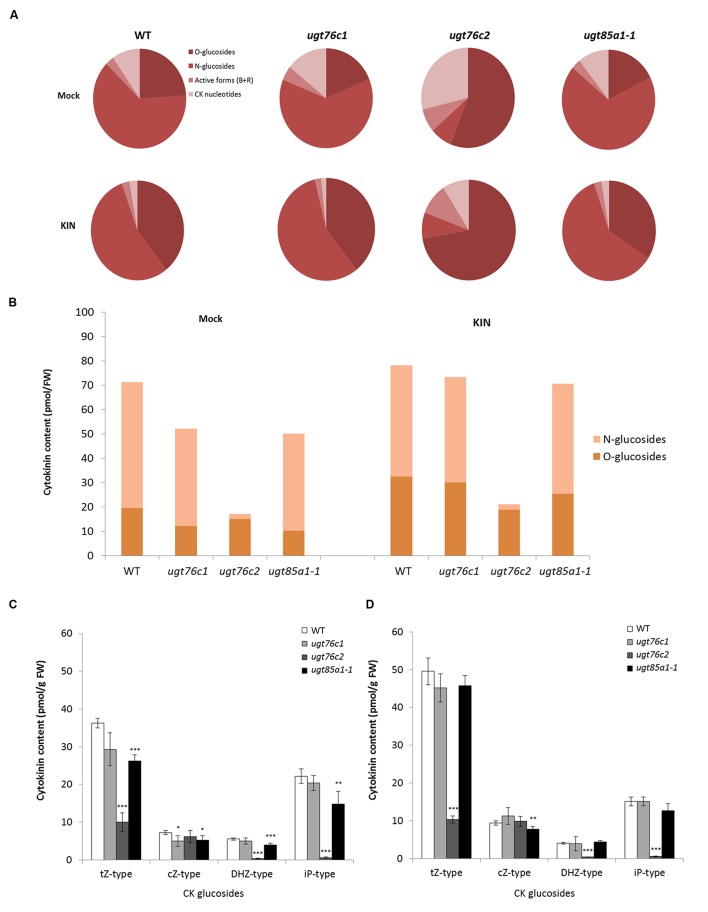
**Cytokinin content in detached leaves of *ugt76c1*, *ugt76c2* and *ugt85a1-1* mutants after 10 μM kinetin treatment.** Ratio among CK forms in mock treatment and KIN treated leaves **(A)**. Distribution of total amounts of CK *O*- and *N*-glucosides in the genotypes **(B)**. Cytokinin content by CK types; each individual CK-type group sums up free base and riboside with their corresponding *O*-glucosides and *N*-glucosides plus ribotides; values are the means of three biological replicates with error bars representing standard deviations. Asterisks over bars indicate significant difference according to two-tailed unpaired Student’s *t*-test, ^∗^*P* < 0.05, ^∗∗^*P* < 0.01, ^∗∗∗^*P* < 0.001 where for each replicate sixth and seventh green fully developed leaves from ten independent 28-day-old rosettes were pooled for control mock treatment **(C)** and KIN treatment **(D)**.

**FIGURE 7:**
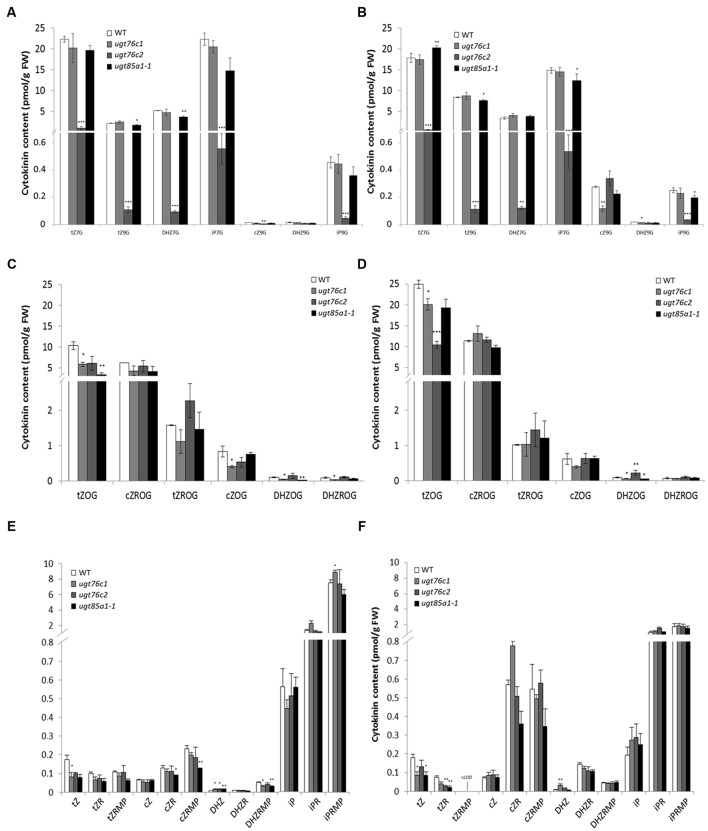
**Detailed cytokinin content in detached leaves of WT, *ugt76c1*, *ugt76c2* and *ugt85a1-1* mutants.** *N*-glucosides content in control mock treatment **(A)** and after KIN treatment **(B)**, *O*-glucosides content in control mock treatment **(C)** and after KIN treatment **(D)**, and CK free bases together with their ribosides and ribotides in control mock treatment **(E)** and after KIN treatment **(F)**. Values are the means of three biological replicates with error bars representing standard deviations. Asterisks over bars indicate significant differences between wild-type and the mutants according to two-tailed unpaired Student’s *t*-test, ^∗^*P* < 0.05, ^∗∗^*P* < 0.01, ^∗∗∗^*P* < 0.001.

Based on our knowledge of plant ability to restore impaired CK homeostasis by modulation of gene expression (Mrízová et al., 2013; Pospíšilová et al., 2016) and of the fact that only minor regulation effect was detected in *ugt76c1* mutant (Wang et al., 2013), we performed an experiment on detached leaves to uncouple the leaf response from whole plant level. We further treated the leaves with exogenously applied KIN to cease CK biosynthesis, thereby boosting visibility of plant’s response to applied CK with focus on glucosylation. Subsequently, we analyzed CK content of all the CK-specific *UGT* loss-of-function mutants in response to exogenously applied KIN. In order to compare the CK content of WT and *ugts* in general, we grouped CK by forms (active, nucleotides, *O*- and *N*-glucosides) and compared their overall ratios as illustrates **Figure 6A**. Here it can be noticed that *ugt76c1* and *ugt85a1-1* shared a similar pattern of CK forms distribution in general, whereas *ugt76c2* showed significantly altered ratio caused mainly by decreased *N*-glucosides portion. *N*-glucosides were in fact almost depleted in *ugt76c2* as shown in **Figure 6B** (comparing total glucosides amounts) whereas they were just moderately decreased in *ugt76c1* and *ugt85a1-1*. **Figure 7B** also shows a portion of *O*-glucosides that are decreased in all the mutants and particularly in *ugt76c1* and *ugt85a1-1* under non-treated condition. Interestingly, higher amounts of *O*-glucosides were detected after KIN uptake in contrast to non-treated leaves in all genotypes, with the lowest amounts in *ugt76c2*. When CK glucosides are presented as individual CK types in absolute numbers (**Figures 6C,D**) it can be concluded that the UGTs are not specific for any particular CK type since glucosides of all CK types were decreased in all the three analyzed mutants. **Figure 7** describes the contents of all individual CK in detail. Neither *ugt76c2* nor *ugt85a1-1* showed any specificity for *N*7- or *N*9- glucosylation since both groups of glucosides were decreased (**Figures 7A,B**) which is in accordance with previous data (Wang et al., 2011, 2013). However, KIN treatment of *ugt76c1* revealed significantly lower level of *c*Z9G (**Figure 7B**) that was not determined in previous studies. Major contributors of total *O*-glucosides levels are *t*ZOG and *c*ZROG. We detected significantly decreased *t*ZOG levels generally in all the mutants under both conditions, whereas the levels of *c*ZROG stayed steady (**Figures 7C,D**). Interestingly, lower levels of *c*ZOG were measured in *ugt76c1* under both conditions. The levels of active CK and their biosynthetic forms are in accordance with our previous experiment with KIN treatment since lower levels of iP and iPRMP were detected after KIN uptake in general as a result of proposed abolished CK biosynthesis (**Figure 7F**). Lower levels of *t*Z under non-treated condition together with lower levels of *t*ZR and depleted *t*ZRMP levels after KIN uptake were assessed in all the mutants, pointing to the plant’s need to restore the homeostasis caused by impaired glucosylation, especially when more stress caused by excessive supply of exogenous CK was applied.

### Cytokinin Metabolism Gene Expression Profile in *ugt* Mutants

**FIGURE 8:**
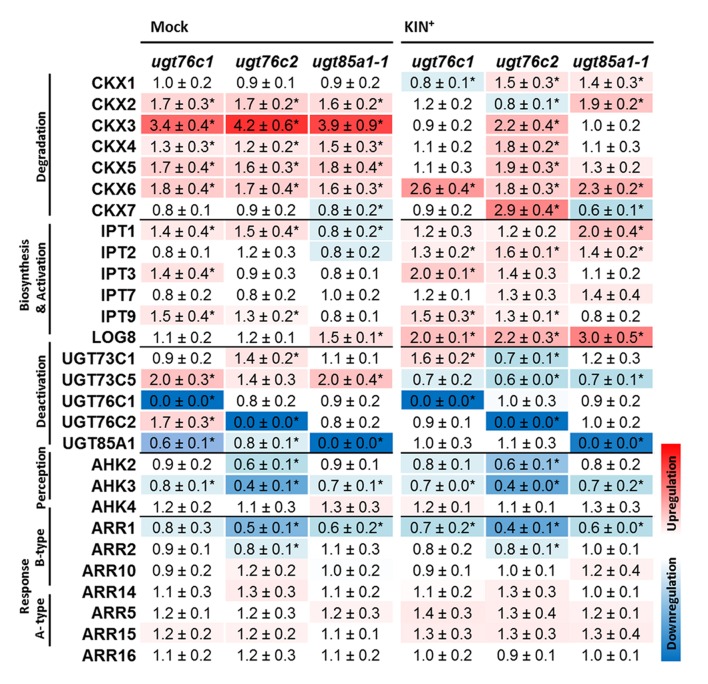
**Expression profiles of cytokinin metabolism, perception and response genes under standard conditions (Mock treatment) and after kinetin treatment (KIN^+^) in detached leaves of *A. thaliana ugt76c1*, *ugt76c2* or *ugt85a1-1* mutants.** The gene expressions of the mutant plants are expressed as relative quantities and extrapolated relative to the mock treatment and KIN treated WT, respectively, given as 1.0. The values represent means of three biological replicates (three technical replicates) with standard deviations. Asterisks indicate significant differences between controls and samples according to two-tailed unpaired Student’s *t*-test, ^∗^*P* < 0.05; < LOD, below limit of detection. No expression was detected for *LOG2*, *LOG6* and *LOG7*.

Gene expression of major CK metabolic pathways, perception and response was analyzed in detached leaves of *ugt* mutants after KIN treatment as well as in control mock treatment. **Figure 8** illustrates RQ of the gene transcripts in a heat map. Here, expression of majority of degradation pathway genes was increased in all the mutants, particularly in non-treated group and specifically also in *ugt76c2* after KIN uptake. The most upregulated form was *CKX3* followed by *CKX5* and *CKX6*. A different pattern was detected for *CKX1* since this isoform was downregulated in *ugt76c1* mutant after KIN uptake, whereas upregulated in the remaining two mutants. Similarly, *CKX2* transcript level was specifically increased in *ugt85a1-1* mutant, but slightly suppressed in *ugt76c2* and not modulated in *ugt76c1. CKX7* form was slightly downregulated in *ugt85a1-1* in both conditions but upregulated in *ugt76c2* after KIN uptake. CK biosynthesis was generally only modestly decreased when not treated by CK with mutant dependent exceptions. *IPT1* and *IPT9* transcripts were more abundant in *ugt76c1* and *ugt76c2*, whereas transcript level of *IPT3* was increased specifically in *ugt76c1* mutant. Majority of *IPT* genes were slightly elevated after KIN treatment when compared to WT. Although *IPT3* and *IPT9* shared a somewhat similar trend as when not treated, *IPT1* behaved in an opposite way in *ugt85a1-1* mutant. Expression of *LOG8* increased in order WT < *ugt76c1* < *ugt76c2* < *ugt85a1-1* both, in plants not treated and treated with KIN, but the increase was more pronounced after the treatment. *UGT85A1* was downregulated in *ugt76c1* and *ugt76c2* mutants when not treated, and *UGT76C2* increased in *ugt76c1*. *AHK3* transcript decreased in all the mutants in both conditions as well as *ARR1*with significance for *ugt76c2*. *AHK2* was specifically decreased in both *ugt76c* mutants after KIN uptake. Two out of three examined A-type *ARR* were upregulated in response to KIN.

### Subcellular Localization of UGTs

**FIGURE 9:**
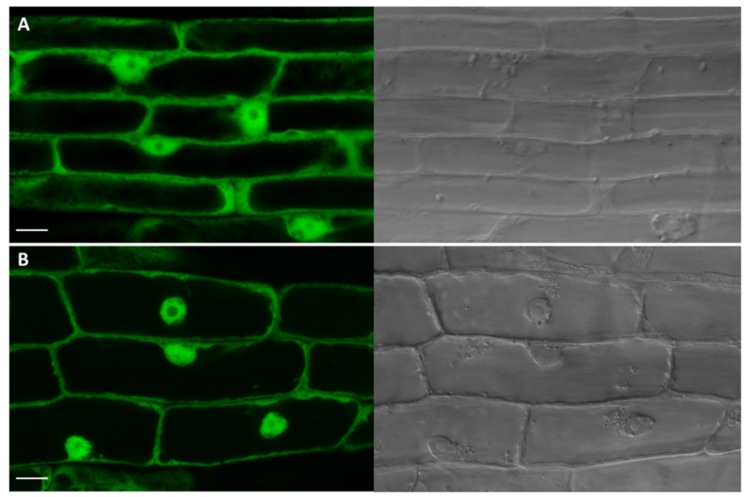
**Subcellular localization analysis of UGT76C2-GFP fusion protein.** Green channel and transmission light images captured by confocal microscopy. **(A)** A control root overexpressing SU:*GFP* with typical GFP pattern in cytosol and nuclei. **(B)** The root cells overexpressing SU:*UGT76C2-GFP* with signal indicating the cytosolic localization of UGT76C2. Scale bars 10 μm.

The only CK-specific UGT with confirmed subcellular localization is UGT85A1 detected in cytosol (Jin et al., 2013). That was in strong contrast with its *in silico* prediction by WoLF PSORT prediction tool^3^ (Woo et al., 2007). In that study, UGT85A1 was predicted as part of the AtUGT85A-(sub)class to be a membrane-associated enzyme, with targeting to chloroplasts and endoplasmic reticulum (ER). The authors further showed that the AtUGT85A1 protein contains the xKQxxEF motif for microsome retention and ER membrane retention signal QKSQ at the C terminus as well as several posttranslational modification sites suggesting high level of posttranslational modification (Woo et al., 2007). However, confirmed localization to cytosol accords with generally accepted presumption that plant UGTs are cytosolic enzymes (Bowles et al., 2005; Sun et al., 2013). Here, we performed a signal peptide prediction of the remaining CK-specific UGTs using available prediction software (**Table 1**). Similar to UGT85A1, UGT76C1and UGT73C1 localization is predicted to chloroplast according to signal peptide prediction analysis. Since UGT76C2 is one of the core CK homeostasis genes, we examined its subcellular localization using confocal laser scanning microscopy using GFP tagging. As shown in **Figure 9B**, the fluorescent pattern of UGT76C2-GFP displayed diffuse fluorescence in cytosol, accumulating along the plasma membrane and in nuclei (Grebenok et al., 1997a,b; Köhler et al., 1997; **Figure 9A**), thus proving UGT76C2 to be a cytosolic enzyme. We suggest the same localization for UGT76C1 based on our signal peptide prediction and comparison (see Supplementary Figure S3).

**Table 1:** Prediction of a signal peptide in protein sequences of UGTs and model proteins.

Protein	TargetP	Prot Comp Plant	SignalP	ChloroP	WoLF PSORT	iPSORT	Plant-mPLoc	Confirmed
UGT73C1	O^5^	Cl^1^	SP^3^	Cl	Cl^3^	Cl^3^	TM	C (unpublished)
UGT73C5	O^3^	Cl TM^2^	SP^3^	–	N M^3^	Cl^1^	TM	C (Husar et al., 2011)
UGT76C1	O^3^	Cl TM^3^	–	–	Cl N^4^	M^3^	TM Cl	C (unpublished)
UGT76C2	O^4^	C^1^	–	–	N^1^	SP^1^	TM	C (this work)
UGT85A1	O^3^	Cl TM^1^	–	Cl	Cl^1^	Cl^1^	TM	C (Jin et al., 2013)
CKX1	M^4^	V^3^	SP^3^	Cl	V^5^	M^4^	V	V (Werner et al., 2003)
CKX2	SP^2^	EC^1^	SP^1^	–	C^2^	–	ER	EC^∗^ (Werner et al., 2003)
CKX7	O^3^	EC^2^	SP^1^	–	C^3^	–	EC	C (Köllmer et al., 2014)
RubSC^†^	Cl^3^	EC^1^	SP^1^	Cl	Cl^1^	Cl^1^	Cl	Cl

*Subcellular compartments: mitochondria (M), chloroplast (Cl), membrane bound protein (TM), unspecified signal peptide (SP), nucleus (N), vacuole (V), cytosol (C), extracellular protein (EC) and other location (O), no signal peptide in the sequence (–). Prediction reliability class (from most reliable to the lowest reliable): 1.000–0.800 (1), 0.800–0.600 (2), 0.600–0.400 (3), 0.400–0.200 (4), 0.200–0.000 (5). ^∗^AtCKX2 is proposed to function in cytosol based on structure and function analogy to ZmCKX1 (Šmehilová et al., 2009). ^†^RubSC, Ribulose bisphosphate carboxylase small chain, functions in ribulose bisphosphate carboxylase complex in CO_2_ fixation localized in chloroplast.*

## Discussion

It is widely accepted that CK levels decrease during leaf senescence; however, this is based on former studies where the information was assumed based only on restricted CK types and forms of only limited time points and plant species analyzed. Specifically, a decreased zeatin level was observed in senescent tobacco leaves (Singh et al., 1992), followed by finding that higher amount of glucosides are present in mature than young tobacco leaves (Benková et al., 1999). In recent studies, CK glucosides were shown to be generally prevalent in the species analyzed so far (Gajdošová et al., 2011). Furthermore, particularly *N*-glucosides were shown to be predominant CK in tobacco (Havlová et al., 2008; Mýtinová et al., 2011; Uzelac et al., 2015). In our experiment, we assessed distribution of CK forms during *Arabidopsis* development with focus on leaf senescence, demonstrating that particularly *N*7- are the most predominant glucosides in *Arabidopsis* under physiological conditions (**Figure 1C**) with enormous accumulation in senescence leaf. Since no particular UGT is responsible for the dramatic increase according to our data, we can therefore assume that the accumulation is caused by fact that the *N*-glucosides are terminal metabolites. Besides *N*-glucosides, *t*ZOG increased specifically in senescent leaf (**Figure 1D**) which agrees with strongly upregulated expression of *UGT85A1* (**Figure 2A**). In senescent leaf, active CK level drops down to approx. 60% of their initial content, which is in strong contrast with more than fivefold increase of CK glucosides. In fact, free bases and ribosides are active in nanomolar concentrations and are maintained within this physiological range ensured by their precise regulation. Thus, this observation raises a question whether CK glucosides, particularly their terminal *N*- forms, possess any physiological role when they accumulate in such enormous concentrations in comparison to active CK. Here it has to be noted that CK *O*-glucosides are not substrates for any maize CKX enzymes (Zalabák et al., 2014), which suggests that glucosylation presumably protects CKs from their rapid degradation. Neither *N*- nor *O*-glucosides were found to be able to trigger known CK receptors (Spíchal et al., 2004; Romanov et al., 2006; Stolz et al., 2011), and no inhibition effect on the receptors was observed for *t*ZOG (Romanov et al., 2005). The question was, whether *N*-glucosides could possibly possess an inhibitory effect on the receptors. In order to extend the current knowledge, we analyzed the most accumulated CK glucosides (iP7G, iP9G, *t*Z7G and *t*Z9G) for their inhibition potency on AHK3 and AHK4 receptors in a competition test (Romanov et al., 2005, 2006) in concentration ratios similar to those detected in senescence stage. However, no inhibition was detected for any of the tested glucosides (data not shown). Therefore we confirmed that glucosylation of CKs has a physiological significance as storage or terminal deactivation mechanism rather than interference of glucosides with the CK signalization pathway.

Up to date, the knowledge of *in vivo* function of UGT85A1 as a CK deactivation enzyme was based on gain-of-function mutants that showed a potential to *O*-glucosylate preferentially *t*Z. Our results showed that *UGT85A1* is specifically expressed in senescence leaves. Since characterization of a loss-of-function mutant is one of the most powerful approaches commonly used to directly unravel the studied protein’s function we used this approach to investigate possible significance of UGT85A1 in senescence. We characterized a true loss-of-function mutant of *UGT85A1* in the present work and extended the knowledge of its proposed specific *O*-glucosylation of *t*Z (Jin et al., 2013) by observation that this isoform could partially contribute also to other CK-glucosides formation (**Figure 5**) if this fact is not caused by other regulatory processes in the mutant. The suggested broader range of CK acceptors of UGT85A1 could be supported by the knowledge that UGTs can glycosylate a rather wider range of substrates (Bowles et al., 2005) and further by the fact that a recombinant UGT85A1 showed comparable specificity to *t*Z and *c*Z substrate as well as to DHZ (Hou et al., 2004). Since detected lower CK**-glucosides concentrations of *ugt85a1-1* did not manifest in phenotype changes in comparison to *ugt76c1* (Wang et al., 2013), we performed experiment with induced senescence in detached leaves and observed decrease in chlorophyll degradation together with enhanced anthocyanin accumulation, further supported by modulated expression of stress and senescence related genes (**Figure 4**). Taken together, our results suggest that UGT85A1 plays important role in proper senescence process progress in *Arabidopsis* leaf.

Former studies pointed out that UGT76C2 could possess a significant role in CK regulation. *UGT76C2* transcript was shown to be upregulated in response to exogenously applied iP (Motte et al., 2013), KIN (Mik et al., 2011) or BAP (Bhargava et al., 2013; Vylíčilová et al., 2016), whereas no regulation of *UGT76C1* or *UGT85A1* was detected. This led us to hypothesis that UGT76C2 might play a prominent role in CK homeostasis maintenance. No difference in expression level of *UGT76C1* and *UGT85A1* can be explained by the fact that BAP, KIN or iP are not the best substrates for the above two enzymes (Hou et al., 2004). Therefore, we performed a comparative study where we assessed an expression profile of individual *UGTs* with all the above substrates implementing *t*Z. Our results showed only slight response of *UGT85A1* to *t*Z uptake, but more importantly, they indicated enormous upregulation of *UGT76C2* expression in response to each of the tested CK (**Figure 2B**). The slight increase in *UGT85A1* transcript after *t*Z uptake was further confronted with publicly available transcriptomic data (not shown here), but no significant response was observed in analyzed experiments. Brenner et al. (2012) performed a comparative meta-analysis of the above transcriptomic experiments and proposed *UGT76C2* to be part of CK response core genes. That is in good agreement with data published by research group of Professor Hou who demonstrated that CK homeostasis is maintained by regulation on CK perception and response level in *ugt76c2* mutant (Wang et al., 2011). Although the same pattern of regulation was shown in *ugt76c1* mutant under standard conditions, the differences in relative quantity in comparison to WT are rather moderate (Wang et al., 2013). Moreover, when comparing CK glucosides content of all the UGTs mutants under standard conditions and after KIN uptake (**Figures 7A–F**), *ugt76c2* possesses the lowest concentrations. Thus, taking into account all our findings together with previously published data, we conclude that UGT76C2 possesses an exclusive role in overall CK homeostasis maintenance.

Besides UGT76C2, fast response to CK treatment is ensured by CKX whose transcripts rise as early as 15 min after the treatment in our previous studies (Mik et al., 2011; Motte et al., 2013). In our experiment with exogenously applied CK, we compared CK content after *t*Z and KIN treatment and found out that, surprisingly, except expected *N*-glucosides formation, *t*ZOG accumulation was also detected in both treatments. Although UGT85A1 was shown to be the main contributor to *t*ZOG pool (Jin et al., 2013), we did not detect any activation on transcript level. It could be hypothesized that the enzyme is activated on protein level or can simply produce more *t*ZOG when needed. In addition, it can be hypothesized that UGT76C2 contributes to increased *t*ZOG level, since we detected decreased levels of *t*ZOG in *ugt76c2* after KIN uptake by detached leaves. Further, it has to be noted that even though KIN was not detected to be a suitable substrate for recombinant UGT85A1 (Hou et al., 2004), upregulated expression of *UGT85A1* was detected after KIN treatment *in vivo* when incubated in continuous dark (Mik et al., 2011). The ratio of *t*Z and *t*ZOG in *ugt76c2* suggests importance of maintenance of adequate *t*ZOG level when UGT76C2 capability of CK glucosylation is impaired.

Our results on CK content determination revealed a significantly different regulation in *ugt76c2* mutant, whereas *ugt76c1* and *ugt85a1-1* shared a similar CK profile with minor differences. The data suggest *UGT76C1* to be more *c*Z specific, which is not in contrast with previous study (Wang et al., 2013). Although we did not conclude any specific physiological function of UGT76C1 in our study in contrast to UGT76C2 and UGT85A1, we detected slightly higher expression of *UGT76C2* in *ugt76c1*mutant. This could eventually explain only slightly decreased glucosides in this mutant in our experimental condition and thus point to speculative co-expression of these closely related isoforms. Yet, since we did not detect any redundancy of these two isoforms in our experiment, the role of UGT76C1 remains unclear. Gene profiling of CK metabolism brought more light to regulatory mechanism of the plant’s need to balance the impaired glucosylation. We confirmed the mechanism of enhanced CK degradation and decreased CK perception (ensured particularly by CKX3, CKX6, AHK2, AHK3 and ARR1) as general when CK glucosylation is impaired, since we observed this pattern in *ugt85a1-1* mutant as well as in *ugt76c1* and *ugt76c2*, as was described before (Wang et al., 2011, 2013). The plants’ enhanced CKX expression agrees with observed decreased levels of *t*Z (**Figures 7E,F**) since it is one of the best substrates of these isoforms (Galuszka et al., 2007). CKX3 was also shown to be the main isoform responsible for CK excesses degradation after KIN uptake in WT *Arabidopsis* (Mik et al., 2011). We detected a specific regulation in individual UGT mutants mediated by CKX1 (in case of *ugt76c2*) and further by CKX2 and CKX7 (in case of *ugt85a1-1* and *ugt76c2*). A specific regulation was also detected in *ugt85a1-1*, since this was the only case when downregulation of CK biosynthesis was observed under standard condition (**Figure 8**). The specific regulation on transcript level goes well with CK content determined based on the enzymes substrate preferences (Miyawaki et al., 2006; Galuszka et al., 2007; Kuroha et al., 2009) and likewise correlates well with previous studies on CKX overexpressing lines (Köllmer et al., 2014). Interestingly, we reported increased levels of *c*Z(R) in *ugt76c2* after KIN uptake (**Figure 7F**) and, reversely, lowered *c*Zs in *ugt85a1-1* in whole plants (**Figure 5**) as well as in detached leaves (**Figure 7F**). Increased levels of *c*Z(R)s were found in many plant species in response to both, abiotic and biotic stress (Schäfer et al., 2015), as well as after exogenous application of CK (Vyroubalová et al., 2009). This phenomenon can support the importance of UGT76C2 action and thus increased *c*Z(R)s accumulation when the action of the protein is impaired. Reversely, lowered *c*Zs could be an accompanying effect of possibly better stress performance of *ugt85a1-1*, especially when taking into account that that lowered level of ABA biosynthetic gene was detected in detached leaves in the *UGT85A1* loss-of-function mutant in our stress experiment.

**FIGURE 10:**
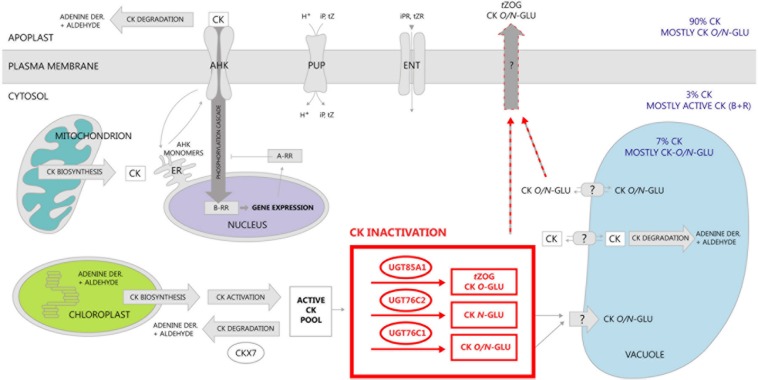
**Schematic distribution of CK and compartmentation of CK metabolism in *Arabidosis*.** CK biosynthesis was localized mainly to plastids and further to mitochondrion and cytosol (Kasahara et al., 2004) followed by their activation that occurs within cytosol (Chen and Kristopeit, 1981a,b; Kuroha et al., 2009). Distribution of distinct CK forms within cellular compartments was elucidated in our recent publication (Jiskrová et al., 2016). Almost no active CK content in apoplast in comparison to CK glucosides is given by the fact that CK degradation takes place prevalently there (Werner et al., 2003). Besides CK degradation in apoplast, two of its enzymes were localized in vacuoles (Werner et al., 2003), whereas only one enzyme (CKX7) was revealed to play its role in cytoplasm (Köllmer et al., 2014). In this study, we confirmed localization of CK glucosylation in cytosol, thereby suggesting a transport mechanism of CK glucosides across plasma membrane out of the cytosol. The only known transporters of CK up to date are purine permeases (PUP) transporting iP and *t*Z (Gillissen et al., 2000; Bürkle et al., 2003; Cedzich et al., 2008), and equilibrative nucleoside transporters (ENT) (Sun et al., 2005; Hirose et al., 2007). Perception and signal transduction of CK was described in many works up to now (for review, see Kakimoto, 2003; or Spíchal, 2012). In this scheme, we adopt a hypothesis from our review (Zalabák et al., 2013) where recycling of AHK monomers between endosomes and plasma membrane (Dortay et al., 2008; Lomin et al., 2011) is proposed as a possible compromise scenario.

In this study, we further investigated compartmentation of CK glucosylation. The fact that UGT76C2 possess an exclusive role in CK homeostasis maintenance raised a question of its subcellular localization that could elucidate compartmentation of CK action. We localized this enzyme using GFP-tagging in cytosol. Analogically to the results obtained for UGT76C2, our results on signal peptide homology and *in silico* prediction for UGT76C1 suggest cytosolic localization of this isoform. UGT85A1 was confirmed to be a cytosolic enzyme before (Jin et al., 2013). As discussed in an outstanding review by Lim and Bowels, glycosylation and deglycosylation can regulate levels of metabolites in pathways through controlling their exit and re-entry from cytosol into their reaction environments (Lim and Bowles, 2004). Based on our newly gained knowledge of CK glucosylation taking place in cytosol, along with the current finding of CK forms compartmentation in *Arabidopsis* (Jiskrová et al., 2016), we present a new hypothesis of CK metabolism compartmentation on subcellular level schematically illustrated in **Figure 10**. We employed former schemes of exquisite reviews (Kasahara et al., 2004; Frébort et al., 2011; Ha et al., 2012; Spíchal, 2012; Zalabák et al., 2013) and supplemented them with all currently known information on CK metabolism compartmentation. Among all *Arabidopsis* CKX enzymes, CKX7 possesses a prominent role in CK homeostasis maintenance inside the cell, since it is the only cytosolic CKX (Köllmer et al., 2014). Interestingly, this is the only CKX isoform with a rather constitutive expression pattern (Mik et al., 2011; Motte et al., 2013). Further, when taking into account kinetic parameters of CKX and UGT enzymes in general (Bilyeu et al., 2001; Hou et al., 2004), we propose that CK glucosylation within the cytosol might serve as an immediate fine-tuning mechanism of inner CK homeostasis that subsequently contributes to overall CK metabolism status once the formed CK-glucosides are accumulated in extracellular space (Jiskrová et al., 2016). Since glycosylation makes molecules more hydrophilic and better accessible to membrane-bound transporters (Lim and Bowles, 2004), we further hypothesize a transport mechanism of CK-glucosides out of the cytosol with specificity to *t*ZOG. In fact, glucosides can enter other more hydrophilic compartments such vacuoles, which was proved for CK-glucosides except *t*ZOG (Jiskrová et al., 2016). Apart from the CK non-specific transport mediated by PUP and ENT transporters across plasma membrane (Gillissen et al., 2000; Bürkle et al., 2003; Hirose et al., 2005, 2007; Sun et al., 2005), not much is known about the CK transport mechanism in general; thus, we emphasize importance of elucidation of CK inter-compartmental transport to overall understanding of CK signalization.

## Conclusion

In this study, we present results of our comprehensive targeted analysis of CK metabolites and related transcript levels of mutants of three CK-specific UGTs. We confronted our results on loss-of-function mutants of cytokinin-specific glycosyltransferases with previous studies and propose physiological significance rather of cytokinin glycosylation process than of CK-glucosides themselves. We hypothesize a quick fine-tuning effect of cytokinin-specific glycosyltransferases on active CK levels in cytoplasm alongside their rapid degradation by CKX enzymes that take place predominantly in extracellular space in *Arabidopsis*.

## Author Contributions

MŠ served as principal investigator, designed and performed the experiments, conceived the project, drafted and finalized the manuscript. JD assisted with the experiments and data analysis, ON with cytokinin content analysis and TT with confocal microscopy. PG and ON assisted with manuscript preparation and revision.

## Conflict of Interest Statement

The authors declare that the research was conducted in the absence of any commercial or financial relationships that could be construed as a potential conflict of interest.

## Footnotes

1 www.softberry.com

2 http://www.genscript.com/wolf-psort.html

3 http://psort.nibb.ac.jp/

## Abbreviations

BAP: *N*^6^-benzylaminopurine
*c*Z: *cis*-zeatin
*c*Z9G: *c*Z *N*9-glucoside
*c*ZOG: *c*Z *O*-glucoside
*c*ZROG: *c*ZR *O*-glucoside
*c*ZR: *c*Z riboside
*c*ZRMP: *c*ZR monophosphate
DHZ: dihydrozeatin
DHZR: DHZ riboside
DHZOG: DHZ *O*-glucoside
DHZR: *O*-glucoside
DHZ7G: DHZ *N*7-glucoside
DHZ9G: DHZ *N*9-glucoside
DHZRMP: DHZR monophosphate
iP: *N*^6^-(Δ^2^-isopentenyl)adenine
iPR: iP riboside
iP7G: iP *N*7-glucoside
iP9G: iP*N*9-glucoside
iPRMP: iPR monophosphate
Kin: kinetin
*t*Z: *trans*-zeatin
*t*Z7G: *t*Z *N*7-glucoside
*t*Z9G: *t*Z *N*9-glucoside
*t*Zr: *t*Z riboside
*t*ZOG: *t*Z *O*-glucoside
*t*ZROG: *t*ZR *O*-glucoside
*t*ZRMP: *t*ZR monophosphate
UGT: uridine diphosphate glucose glycosyltransferase
