# Supplementary material for: Cytokinin-Specific Glycosyltransferases Possess Different Roles in Cytokinin Homeostasis Maintenance
Source: Front Plant Sci. 2016 Aug 23;7:1264. doi: 10.3389/fpls.2016.01264 (PMC4993776; doi:10.3389/fpls.2016.01264)
Supplement: Supplementary file 1 [file Data_Sheet_1.PDF]

Supplementary Material

Cytokinin-Specific Glycosyltransferases Possess Different Roles in Cytokinin Homeostasis Maintenance

Mária Šmehilová, Jana Dobrůšková, Ondřej Novák, Tomáš Takáč, Petr Galuszka
Correspondence: Corresponding Author: maria.smehilova@upol.cz

Supplementary Tables

Table S1. Genotypes used in this work. Homozygous *Arabidopsis thaliana* T-DNA insertional lines from SALK collection of the European Arabidopsis Stock Center (NACS) were used in this study.

Gene	TAIR ID	T-DNA line ID	Mutant	T-DNA insertion	Published
UGT76C2	At5g05860	SALK_135793C	*ugt76c2*	exon	(Wang et al., 2011)
UGT76C1	At5g05870	SALK_144355C	*ugt76c1*	exon	(Wang et al., 2013)
UGT85A1	At1g22400	SALK_146306C	*ugt85a1-1*	promoter	This work
		SALK_085809C	*ugt85a1-2*	exon	(Carviel et al., 2009)

Supplementary Figures

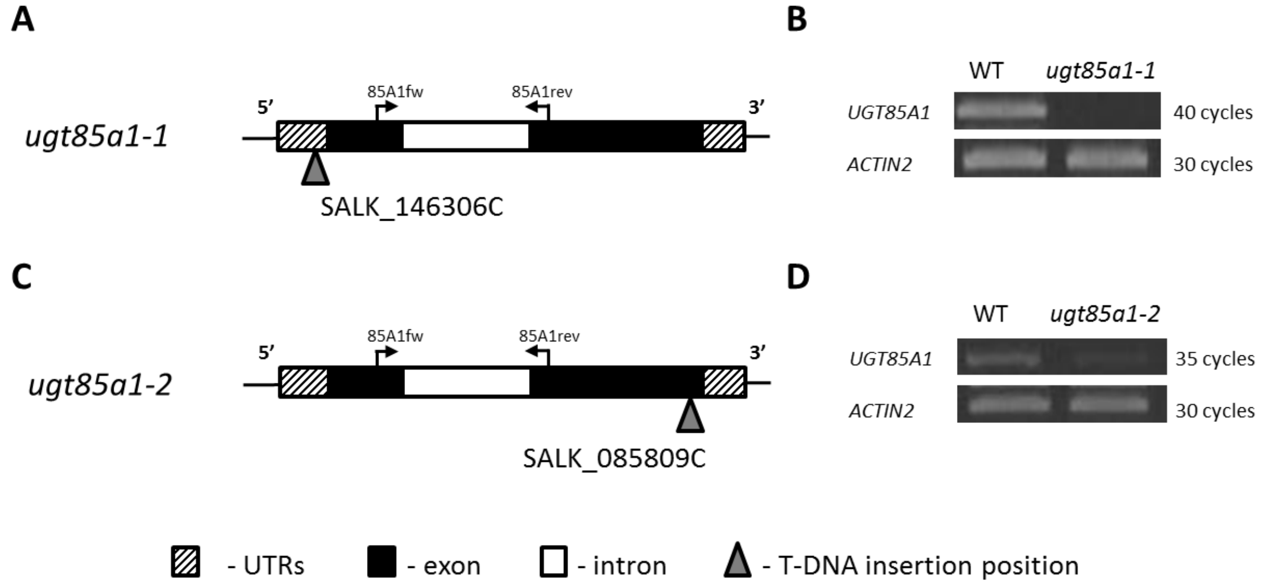

Figure S1. Schematic description of *ugt* T-DNA insertion mutants. Position of T-DNA insertion in *UGT85A1* gene in *ugt85a1-1* **(A)** and *ugt85a1-2* mutant (**C**). Expression assay of *UGT85A1* in *ugt85a1-1* **(B)**, *ugt85a1-2* **(D)** and wild-type (WT).

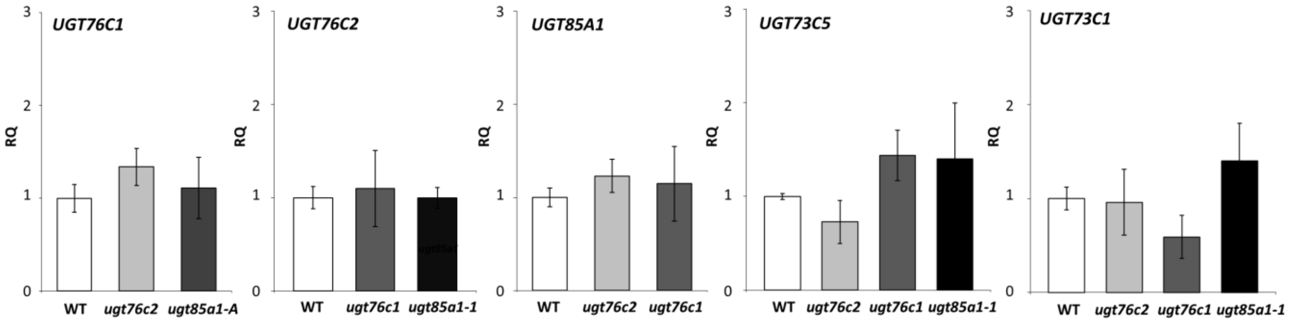

Figure S2. Relative expression levels of cytokinin UGT genes in two-week-old seedlings of wild type and *ugt76c1*, *ugt76c2* and *ugt85a1-1* mutants. The gene expressions are relative quantities extrapolated relative to the WT given as 1.0. Values are the means of three biological replicates with error bars representing standard deviations (No significant difference was detected).

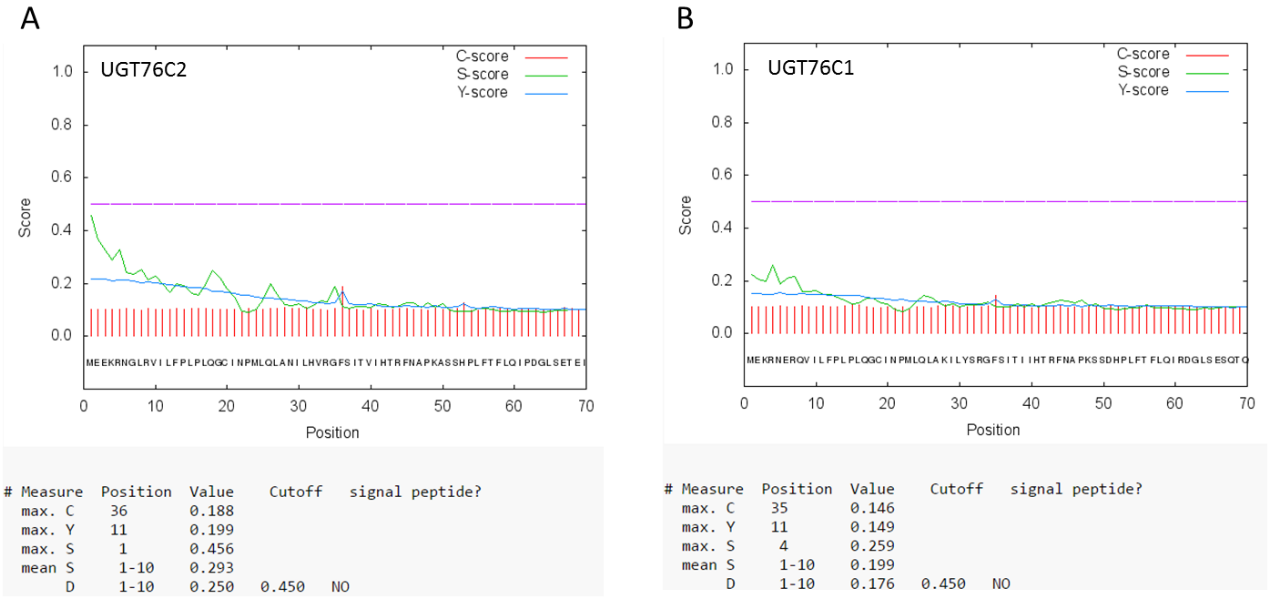

Figure S3. Prediction of subcellular localization of UGT76C2 (A) and UGT76C1 (B). Detailed output of signal peptide prediction by SignalP 4.1 software (Petersen et al., 2011). The plots are amino acid positions of the protein sequences versus prediction scores: C-score (raw cleavage site score); S-score (signal peptide score); Y-score (combined cleavage site score); and mean S (average S-score of the possible signal peptide) with final D-score (discrimination score) that discriminates between signal peptide presence or absence (the lower the scores the higher probability of no signal peptide presence in the protein). Here we demonstrate that UGT76C1 possesses higher probability to not having a signal peptide in comparison to UGT76C2 which we confirmed to be a cytosolic protein using GFP tagging in this study.
